# The rare green‐blue neutrophil inclusion bodies. Worth reporting in blood films? A case of a patient with polytrauma

**DOI:** 10.1002/ccr3.3105

**Published:** 2020-07-16

**Authors:** Rebecca Katrina Bowen, Ioannis Koutsavlis

**Affiliations:** ^1^ Haematology and Blood Transfusion South Lab Victoria Hospital NHS Fife Kirkcaldy UK; ^2^ Haematology NHS Fife Kirkcaldy UK

**Keywords:** hematology

## Abstract

Blood films are an easy tool to aid the diagnosis and management of unwell patients and should be prepared in this setting. Green‐blue neutrophil inclusion bodies could be an ominous sign of poor prognosis in critically ill patients.

## Graphical abstract

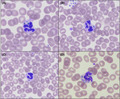

The case is reported of a 51 year old who presented to the emergency department following a fall from a 30 feet high flyover. He had a traumatic cardiac arrest and sustained multiple injuries, pneumothorax, and urethral injury. Chest drains inserted and was intubated and ventilated in the intensive care unit (ICU) following laparotomy and abdominal packing. He returned to the surgical ward following 31 days admission in ICU. Blood films made at day 3 showed green‐blue neutrophil inclusions bodies. (Figure 1) These inclusions are commonly associated with liver injury, critical illness, and/or multiorgan failure. It is usually difficult to find, and a wide scan of a blood film is necessary. Only 1%‐2% of neutrophils may contain these inclusions but also occasionally can be found in monocytes.^1^, ^2^ It is believed that the granules are composed of lipopigments: lipofuscin and ceroid, which may accumulate in the lysosomes of cells in pathological conditions. Our patient survived his critical illness. There is a debate whether these inclusion bodies should be reported as part of the routine blood film. The argument has been early detection may indicate poor prognosis, and therefore, more careful and intensive approach of clinical care would be appropriate. However, the cases reported refer to already unwell patients who are managed to a critical care setting and such a finding in a blood film is not altering the management. Nevertheless, as many cases series with these inclusions report early death, careful monitoring of vital organs and primarily for more severe liver injury is needed.

**FIGURE 1:**
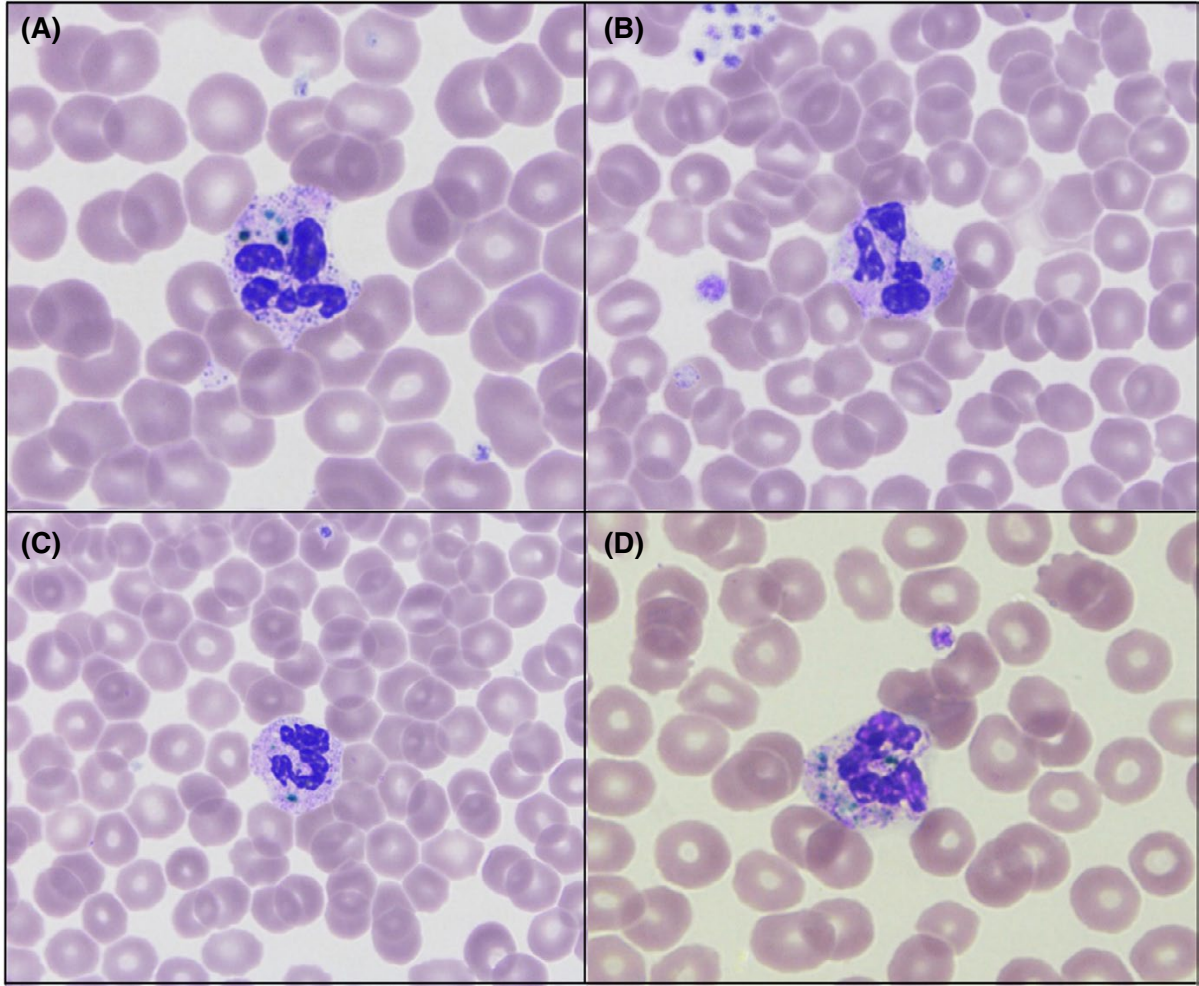
Blood film with the blue‐green neutrophil inclusion bodies at Day 3 of admission following polytrauma. (A‐D) Giemsa‐Wright. Single inclusion (B, C) and multiple inclusions (A, D). Images photographed at 100× magnification

## CONFLICT OF INTEREST

Nothing to report.

## AUTHOR CONTRIBUTIONS

RKB: took the pictures, reviewed, and approved the manuscript. IK: wrote and approved the manuscript.

## ETHICAL APPROVAL

Consent has been obtained from the patient prior to publication.
